# Latent classes of emotional and behavioural problems in epidemiological and referred samples and their relations to DSM-IV diagnoses

**DOI:** 10.1007/s00787-016-0918-2

**Published:** 2016-11-14

**Authors:** Valentina Bianchi, Paolo Brambilla, Marco Garzitto, Paola Colombo, Livia Fornasari, Monica Bellina, Carolina Bonivento, Alessandra Tesei, Sara Piccin, Stefania Conte, Giampaolo Perna, Alessandra Frigerio, Isabella Castiglioni, Franco Fabbro, Massimo Molteni, Maria Nobile

**Affiliations:** 1 Scientific Institute, IRCCS Eugenio Medea, Bosisio Parini, Lecco, Italy; 2 Institute of Molecular Bioimaging and Physiology, National Research Council, Milan, Italy; 3 Department of Neurosciences and Mental Health, Psychiatric Clinic, Fondazione IRCCS Ca’ Granda Ospedale Maggiore Policlinico, University of Milan, Milan, Italy; 4 Department of Psychiatry and Behavioral Sciences, University of Texas Health Science Center at Houston, Houston, TX USA; 5 Scientific Institute, IRCCS Eugenio Medea, San Vito al Tagliamento, Pordenone, Italy; 6 Department of Experimental and Clinical Medicine, University of Udine, Udine, Italy; 7 InterUniversity Center for Behavioral Neurosciences (ICBN), University of Udine, Udine, Italy; 8 Department of Psychology, University of Milano-Bicocca, Milan, Italy; 9 NeuroMI, Milan Center for Neuroscience, Milan, Italy; 10 Hermanas Hospitalarias, FoRiPsi, Albese con Cassano, Italy

**Keywords:** Child Behaviour Checklist, Dysregulation, Latent class analysis (LCA), Psychopathology, Childhood, Adolescence

## Abstract

Researchers’ interest have recently moved toward the identification of recurrent psychopathological profiles characterized by concurrent elevations on different behavioural and emotional traits. This new strategy turned to be useful in terms of diagnosis and outcome prediction. We used a person-centred statistical approach to examine whether different groups could be identified in a referred sample and in a general-population sample of children and adolescents, and we investigated their relation to DSM-IV diagnoses. A latent class analysis (LCA) was performed on the Child Behaviour Checklist (CBCL) syndrome scales of the referred sample (*N* = 1225), of the general-population sample (*N* = 3418), and of the total sample. Models estimating 1-class through 5-class solutions were compared and agreement in the classification of subjects was evaluated. Chi square analyses, a logistic regression, and a multinomial logistic regression analysis were used to investigate the relations between classes and diagnoses. In the two samples and in the total sample, the best-fitting models were 4-class solutions. The identified classes were Internalizing Problems (15.68%), Severe Dysregulated (7.82%), Attention/Hyperactivity (10.19%), and Low Problems (66.32%). Subsequent analyses indicated a significant relationship between diagnoses and classes as well as a main association between the severe dysregulated class and comorbidity. Our data suggested the presence of four different psychopathological profiles related to different outcomes in terms of psychopathological diagnoses. In particular, our results underline the presence of a profile characterized by severe emotional and behavioural dysregulation that is mostly associated with the presence of multiple diagnosis.

## Introduction

The Child Behaviour CheckList/6–18 (CBCL/6–18) [1] is a parent report form to screen for emotional, behavioural, and social problems in children and adolescents aged 6–18. It is an empirically based questionnaire, and its reliability and validity have been widely demonstrated [1]. The checklist returns a profile of scores on eight syndrome scales and six DSM-oriented scales. Several studies have examined the associations between CBCL/6–18 scales and diagnoses based on DSM criteria [2, 3]. In recent years, however, researchers’ interests have moved toward the identification of recurrent profiles characterized by concurrent elevations on more scales, and among these profiles one that has received particular attention is the Child Behaviour Checklist-Dysregulation Profile (CBCL-DP) [4]. This profile is characterized by co-occurring elevations in attention problems, aggressive, and anxious/depressed scales, and it reflects the condition of emotional and behavioural dysregulation described for the first time by Biederman et al. [5]. The CBCL-DP has demonstrated its utility in terms of diagnosis and outcome prediction [4, 6–10]. The majority of these studies used an a priori identified profile that sums the T-score of the three scales, or they alternatively used person-centred statistical approaches, such as Latent class analysis (LCA), applying the method exclusively to the items of these identified three scales. Only a small number of studies used a bottom-up approach, which explores the different profiles that emerge within a population while considering all the syndromic scales of the CBCL. This approach can be found in the work of Connell et al. [11] and of Basten et al. [12]. These studies used LCA to identify profiles of psychopathological traits from CBCL/1.5–5 scales in a sample of economically disadvantaged families and in a population-based sample. Despite coming from different starting points, both authors identified a 4-class solution with an externalizing class, an internalizing class, a normative group, and a highly problematic and dysregulated class. Only one study [13] applied this methodology on a sample of older children and adolescents. These authors performed an LCA on CBCL/6-18 syndrome scales of a combined sample of general-population subjects and referred children and they identified six classes of subjects, including a dysregulated class. The main limit of this interesting work, however, is the small sample size. Interestingly, Bonadio et al. [14] in a similar way used Latent Profile Analysis (i.e. LCA with continuous indicators) to identify psychopathological profiles from the Ohio Scales-Problem Severity Scale (OS PS) filled out by youth and parent dyads of a community mental health sample. In this case, using a different instrument, authors identified five classes of subjects, including a high risk class with elevated scores both on internalizing and externalizing symptoms, which very closely resemble the CBCL-DP.

The present study had two major aims. The first aim was to replicate and extend the preliminary results presented by De Caluwé et al. [13] by examining whether it was possible to identify different psychopathological profiles in a very large mixed sample (including both general and referred children and adolescents). To achieve this goal, we performed an LCA, including the full spectrum of the CBCL syndromic scales. The second aim was to evaluate the relationship between identified classes and DSM-IV diagnoses, also taking the presence/absence of comorbidity into consideration.

## Methods

### Subjects

To maximize the population variability in all scales, a combined sample was used that included both general-population subjects and referred subjects.

*The Italian preadolescent mental health project* (*PrISMA*) is a two-phase survey that was carried out in Italy to estimate the prevalence and correlates of mental health problems in urban preadolescents. The study population consisted of 3418 subjects at the time of the first screening phase (49.6% males; 10–14 years old, *M* = 12.08, SD = 0.90) and of 631 subjects in the second diagnostic phase (46.0% males; 10–14 years old, *M* = 12.16, SD = 0.91). The main features of the *PrISMA* study are summarized in Table 1. Full details concerning research design and methods are available elsewhere [15, 16].

**Table 1:** Socio-demographic and behavioural characteristics of the samples

	PrISMA	Genesis	Total sample
Socio-demographic characteristics			
N°	3418	1225	4643
Male (*n* %)	1695 (49.6%)	942 (76.9%)	2637 (56.8%)
Age (mean ± SD)	12.08 ± 0.90	9.11 ± 2.34	11.30 ± 1.94
Mother education at risk (*n* %)	872 (25.5%)	456 (37.2%)	1328 (28.6%)
Father education at risk (*n* %)	1011 (29.6%)	515 (42.0%)	1526 (32.9%)
Frequencies of syndrome scales score in the clinical range (*n* %)			
Anxious/depressed	494 (14.5%)	489 (39.9%)	983 (21.2%)
Withdrawn/depressed	409 (12.0%)	428 (34.9%)	837 (18.0%)
Somatic complaints	373 (10.9%)	187 (15.3%)	560 (12.1%)
Social problems	308 (9%)	431 (35.2%)	739 (15.9%)
Thought problems	263 (7.7%)	298 (24.3%)	561 (12.1%)
Attention problems	382 (11.2%)	598 (48.8%)	980 (21.1%)
Rule-breaking behaviour	69 (2.0%)	207 (16.9%)	276 (5.9%)
Aggressive behaviour	240 (7.0%)	412 (33.6%)	652 (14.0%)

The *Genesis* project is an ongoing longitudinal study on a clinical sample of children and adolescents who were referred for emotional and behavioural problems to the Child Psychiatry Unit of ‘Eugenio Medea’ Scientific Institute in Bosisio Parini (LC), Conegliano Veneto (TV), Pasian di Prato (UD), and San Vito al Tagliamento (PN). The subsample used in this study consisted of 1226 subjects during the first assessment (76.9% males; 6–17 years old, *M* = 9.11, SD = 2.34). The main features of the *Genesis* study are summarized in Table 1.

The study protocols were approved by the Research Ethical Committee of our Scientific Institute and have been performed in accordance with the ethical standards laid down in the 1964 Declaration of Helsinki and its later amendments. Parents’ written informed consent was obtained for all participants.

### Measures

#### Socio-economic status

For this study, we selected fathers’ and mothers’ educational levels as an indicator of socio-economic status. The parents’ education levels were recoded for analysis into two classes: ‘at risk’ (less than 10 years of school) and ‘not at risk’ (10 years or above). Data of the two separate samples and of the total sample are reported in Table 1.

#### Emotional and behavioural assessment

*Child Behaviour CheckList* 6–18 (CBCL/6–18) [1]. This is an empirically based checklist of social competence and behavioural problems that was filled out by parents of children and adolescents aged 6–18. According to the Achenbach System of Empirically Based Assessment (ASEBA), the CBCL/6–18 is divided into eight syndrome scales: Anxious/depressed, Withdrawn/depressed, Somatic complaints, Social problems, Thought problems, Attention problems, Rule-breaking behaviour, and Aggressive behaviour. In this study, we used the T-score based on the set of multicultural norms ‘group 2’, which applies to the normative sample of the Italian population [17, 18]. Scores on the eight clinical scales were dichotomized as ‘not at risk’ (T < 65) or ‘at risk’ (T ≥ 65). Clinical characteristics of the samples are presented in Table 1.

*Development and Well*-*Being Assessment* (DAWBA) [19]. This is a diagnostic interview that combines a structured and a semi-structured part and is designed to generate the present-state psychiatric diagnoses for children and adolescents following DSM-IV criteria. The DAWBA has shown satisfactory validity and inter-rater reliability [19]. This interview was administered to the 631 subjects of the second phase of the *PrISMA* project. The diagnostic evaluation was conducted on probable cases of mental disorders and on a sample of non-probable cases. All subjects exceeding the cutoff scores (90th percentile of the frequency distribution) of CBCL internalizing and/or externalizing scales and a 10% random sample of those who did not exceed the cutoff scores were selected for this second phase. Full details concerning research design and methods are available elsewhere [15].

*Kiddie schedule for affective disorders and Schizophrenia for school*-*age children*—*present and lifetime version* (K-SADS-PL) [20]. This is a semi-structured diagnostic interview created to assess current and past episodes of psychopathology in children and adolescents according to DSM-III-R and DSM-IV criteria. All subjects in the *Genesis* project were assessed through K-SADS-PL interviews.

Clinical and socio-demographic characteristics of subjects who have entered the diagnostic phase are reported in Table 2.

**Table 2:** Clinical and socio-demographic characteristics of the subjects who have entered the diagnostic phase

	PrISMA	Genesis	Total sample
Socio-demographic characteristics			
N°	631	1225	1856
Male (*n* %)	290 (46.0%)	942 (76.9%)	1232 (66.4%)
Age (mean ± sd)	12.16 ± 0.91	9.11 ± 2.34	11.29 ± 1.94
Frequencies of DSM-IV diagnoses (*n* %)			
Any diagnosis	104 (16.5%)	1058 (86.4%)	1162 (62.6%)
Attention-deficit hyperactivity disorder	21 (3.3%)	386 (31.5%)	407 (21.9%)
Any behaviour disorder	13 (2.1%)	162 (13.2%)	175 (9.4%)
Any mood disorder	13 (2.1%)	372 (30.4%)	385 (20.7%)
Any anxiety disorder	77 (12.2%)	537 (43.8%)	614 (33.1%)
Other diagnoses	3 (0.5%)	110 (9.0%)	113 (6.1%)
Presence of comorbidity	34 (5.4%)	451 (36.8%)	485 (26.1%)

### Data analyses

#### Preliminary analysis

To identify differences between the two samples, they were compared using the *χ* ^2^ statistic in terms of gender, parents’ educational levels, CBCL/6–18 scores, and the *T* test for age.

#### Latent class analysis

To examine whether different groups of subjects could be identified in the two samples, we performed a Latent Class Analysis (LCA), a person-centred statistical approach able to assign persons to a statistically independent class when they respond in the same way to items (or scales) of a questionnaire [21]. Thus, each class has a specific symptom (item or scale) endorsement profile [22]. LCA was performed using Mplus 6.11 [23] on the CBCL/6–18 syndrome scales in the two samples separately (with age and gender as covariates) and in the total sample (introducing the clinical status as covariate). Models estimating 1-class through 5-class solutions were compared. The best solution was determined by looking at the Bayesian Information Criterion (BIC) [24], the Lo–Mendell–Rubin test (LMRT) [25] and the Bootstrapped Likelihood Ratio test (BLRT) [26]. In addition to these fit statistics, in determining the number of classes we also considered the rule of parsimony and the substantive relevance of a class [27]. The identified classes were given descriptive labels based on the consensus of the authors after reviews of each class’s unique profile. During the validation phase of latent classes, each subject was assigned to their highest probability class using the ‘knownclasses’ algorithm.

After conducting separate LCAs for the two samples and for the total sample, we evaluated the classification agreement using the Cohen’s Kappa coefficient. The degree of agreement was interpreted according to the magnitude guidelines defined by Landis and Koch [28].

#### Relationship between classes and diagnostic profiles

In this second step of analysis, we used data only from the subjects who have entered the diagnostic phase. We analysed the distribution of diagnoses between classes and we checked for significant differences using the *χ* ^2^ statistics. Diagnoses were re-coded into major diagnostic categories as follows: Attention-deficit Hyperactivity Disorder, Behaviour Disorders (Oppositional Defiant Disorder, Conduct Disorder, and Disruptive Disorder NOS), Mood Disorder (Depressive Disorder, Dysthymic Disorder, and Depressive Disorder NOS), Anxiety Disorder (Generalized Anxiety Disorder, Specific Phobia, Panic Disorder, Social Phobia, Separation Anxiety, Obsessive–Compulsive Disorder, Post-Traumatic Stress Disorder, Mixed Anxiety Depressive Disorder, and other Anxiety Disorders NOS), and Other Diagnoses (all diagnostic conditions that are not an emotional or a behavioural disorder, such as Tic Disorder, Stuttering, Enuresis, and Selective Mutism).

In order better to evaluate the strength of the association between classes and diagnostic status, this was re-coded as 0 = absence of diagnosis, 1 = one diagnosis, and 2 = two or more diagnoses (i.e. comorbidity). We evaluated whether the classes were able to predict the absence of diagnosis with a backward logistic regression. Subsequently, we performed a backward multinomial logistic regression with diagnostic status as the dependent variable (with absence of diagnosis as reference category) and classes as predictors. Age, gender, and parents’ educational levels were entered as covariates.

The likelihood ratio Chi square was used to test the significance (*p* < 0.05) of all models, whereas the Wald statistic was used to test the significance (*p* < 0.05) of the independent variables. For each model, we also reported Nagelkerke pseudo-*R* ^2^ to show each model’s fit. We used the odd ratio (OR) as a measure of effect size and Monson’s classification of OR [29] to describe the strength of the association between the dependent variables and the predictors.

## Results

### Preliminary analysis

The *PrISMA* and *Genesis* samples differ for age (*t* = 43.317, *p* = 0.000), gender distribution (*χ* ^2^ = 272.940, *p* = 0.000), mothers’ and fathers’ educational levels (respectively, *χ* ^2^ = 113.221, *p* = 0.000; *χ* ^2^ = 125.368, *p* = 0.000), and the percentage of subjects in the clinical range (*χ* ^2^ from 15.778 to 765.010, *p* = 0.000), with the *Genesis* sample having younger subjects, a higher rate of male subjects, a higher percentage of subjects with a lower socio-economic status, and a higher percentage of subjects in the clinical range in all CBCL/6-18 scales.

### Latent class analysis

Table 3 presents the model fit indices for 2- to 5-class solutions of the three LCAs. According to BLRT (and to MLRT for the Total sample), more classes resulted in better models fit, while LMRT (with the exception of Total sample) and BIC suggested the 4-class solutions as the best-fitting models. As the 4-class solution resulted in clearly distinct classes, and considering that a comparison of the 4-class ad 5-class solutions showed that the 5-class solution included an additional class with a profile that was not clearly different from that of the lowest scoring class, we choose the 4-class solution in all the samples. The four classes identified in the three LCAs had a very similar structure, with limited differences between the *PrISMA* and *Genesis* samples.

**Table 3:** Fit statistics for latent class models

	Log-likelihood	BIC	LMRT (*p*)	BLRT (*p*)
PrISMA sample				
2 Classes	−6986.79	14128.18	2414.22 (0.00)	2441.19 (0.00)
3 Classes	−6896.90	14037.91	177.19 (0.00)	179.78 (0.00)
4 Classes	−6841.55	14016.71	109.48 (0.00)	110.70 (0.00)
5 Classes	−6811.38	14045.87	29.68 (0.28)	60.35 (0.00)
Genesis sample				
2 Classes	−5192.34	10519.78	1201.48 (0.00)	1216.85 (0.00)
3 Classes	−5119.34	10451.99	144.16 (0.01)	146.01 (0.00)
4 Classes	−5054.25	10400.03	128.54 (0.00)	130.18 (0.00)
5 Classes	−5032.06	10419.34	43.81 (0.09)	44.369 (0.00)
Total sample				
2 Classes	−12442.97	25054.80	5788.68 (0.00)	5845.82 (0.00)
3 Classes	−12149.40	24568.97	581.41 (0.00)	587.14 (0.00)
4 Classes	−11982.00	24335.50	331.52 (0.00)	334.79 (0.00)
5 Classes	−11940.07	24352.95	83.05 (0.01)	83.87 (0.00)

The Cohen’s Kappa coefficient indicated a substantial agreement (*κ* = 0.766; 95% CI, 0.590 to 0.942, *p* < 0.0005) between subject classifications obtained by running separate LCAs on the original samples and on the total sample. In subsequent analyses, we used the classification obtained in the total sample.

**Fig. 1:**
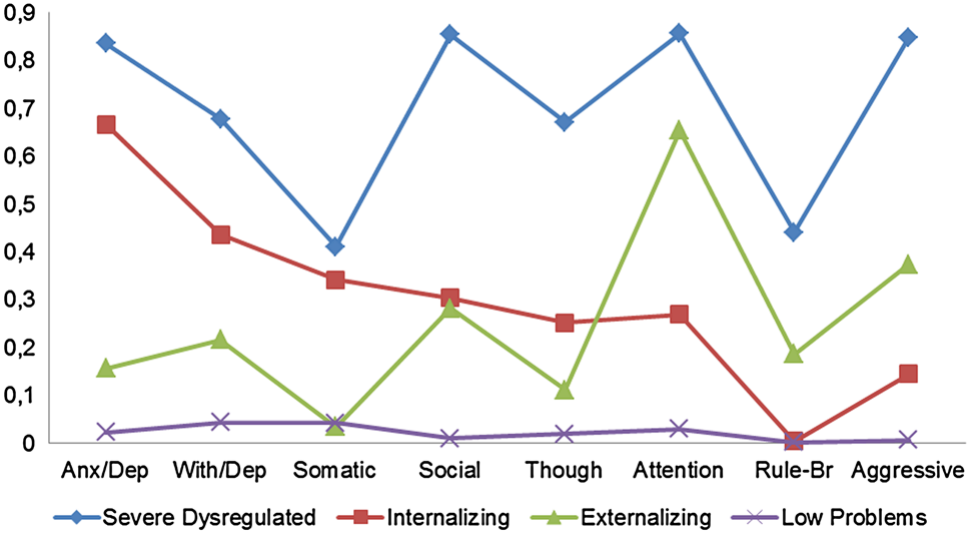
CBCL/6–18 profiles according to the 4-class solution in the total sample

The analysis of the features revealed important differences between classes (Fig. 1). The first class, labelled ‘Severe Dysregulated–DYS’ (7.82% of the sample), includes subjects with an elevated probability (>60%) of being in the clinical range for all CBCL scales, with the exception of Somatic Complaints and Rule-Breaking behaviour. The second class, called ‘Internalizing Problems–INT’ (15.68%), is characterized by a high probability of being in the clinical range only for the Anxious/depressed scale. The third class, ‘Attention/Hyperactivity–ADHD’ (10.19%), has an elevated probability of being in the clinical range for the Attention Problems scale. Finally, the fourth class, labelled ‘Low Problems–LOW’ (66.32%), includes those subjects with a low probability for each CBCL syndrome scale.

### Relationship between classes and diagnostic profiles

Table 4 shows the distribution of diagnostic categories in the four identified classes and related *χ* ^2^ indices. The results of the logistic regression analysis are reported in Table 5. All classes with the exception of ADHD significantly predicted the absence of diagnosis. More specifically, there is a strong association between the absence of diagnosis and the Low Problems class (OR = 8.17; 95% CI 5.61–11.91). There is also a moderate negative association between the absence of diagnosis and the Severe Dysregulated and Internalizing Classes (respectively, OR = 0.58; 95% CI 0.36–0.94; OR = 2.02; 95% CI 1.40–2.98).

**Table 4:** Clinical descriptions of the classes

	Class 1: DYS (339)	Class 2: INT (500)	Class 3: ADHD (416)	Class 4: LOW (601)	*p* value
DSM-IV diagnoses (*n* %)					
Any diagnosis	302 (89.1%)	296 (59.2%)	351 (84.4%)	213 (35.4%)	*χ* ^2^ = 377.667 *p* = 0.000^a, c, d, e f^
ADHD	104 (30.7%)	32 (6.4%)	204 (49.0%)	67 (11.1%)	*χ* ^2^ = 304.967 *p* = 0.000^a, b, c, d, e f^
Any behaviour disorder	64 (18.9%)	16 (3.2%)	66 (15.9%)	29 (4.8%)	*χ* ^2^ = 93.263 *p* = 0.000^a, c, d, f^
Any mood disorder	151 (44.5%)	132 (26.4%)	66 (15.9%)	36 (6.0%)	*χ* ^2^ = 212.112 *p* = 0.000^a, b, c, d, e f^
Any anxiety disorder	184 (54.3%)	214 (42.8%)	99 (23.8%)	117 (19.5%)	*χ* ^2^ = 156.639 *p* = 0.000^a, b, c, d, e^
Presence of comorbidity	177 (52.2%)	144 (28.8%)	100 (24.0%)	64 (10.6%)	*χ* ^2^ = 196.882 *p* = 0.000^a, b, c, e f^

^a^Class 1 vs. class 2 was significantly different (*p* < 0.05)

^b^Class 1 vs. class 3 was significantly different (*p* < 0.05)

^c^Class 1 vs. class 4 was significantly different (*p* < 0.05)

^d^Class 2 vs. class 3 was significantly different (*p* < 0.05)

^e^Class 2 vs. class 4 was significantly different (*p* < 0.05)

^f^Class 3 vs. class 4 was significantly different (*p* < 0.05)

**Table 5:** Significant OR (*p* < 0.05) in the logistic regression and multinomial logistic regression analysis with classes as predictors and age, gender, and parental education as covariates

	Logistic regression	Multinomial regression (absence of diagnosis is the reference category)	
	Absence of diagnosis (*N* = 694)	One diagnosis (*N* = 677)	Comorbidity (*N* = 486)
Severe dysregulated class	0.58** (0.36–0.94)	8.57*** (5.38–13.66)	27.69**** (16.78–45.70)
Internalizing class	2.02** (1.40–2.98)	2.93** (2.08–4.12)	6.63*** (4.43–9.94)
Attention/hyperactivity class	–	8.51*** (5.74–12.60)	7.27*** (4.54–11.65)
Low problems class	8.17*** (5.61–11.91)	–	–
Age	1.55** (1.46–1.65)	0.66** (0.62–0.71)	0.62** (0.58–0.66)
Gender	1.77** (1.37–2.30)	1.79** (1.34–2.37)	1.76** (1.28–2.41)
Mother’s education	0.63** (0.47–0.83)	1.56** (1.16–2.11)	1.65** (1.18–2.29)
Father’s education	–	–	–
Nagelkerke pseudo-*R* ^2^	0.41	0.41	

In brackets 95% CI for significant OR (*p* < 0.05); * weak association, ** moderate association, *** strong association, **** very strong association

The multinomial logistic regression revealed that the ‘One Diagnosis’ condition was significantly associated with the Severe Dysregulated, Internalizing, and Attention/Hyperactivity Classes (respectively, OR = 8.57; 95% CI 5.38–13.66; OR = 2.93; 95% CI 2.08–4.12; OR = 8.51; 95% CI 5.74–12.60 for one diagnosis vs. absence of diagnosis) with a moderate association in the second case and strong associations for the other classes. The ‘Comorbidity’ condition—defined as two or more diagnoses—is significantly associated with the severe dysregulated, internalizing, and Attention/Hyperactivity classes (respectively, OR = 27.69; 95% CI 16.78–45.70; OR = 6.63; 95% CI 4.43–9.94; OR = 7.27; 95% CI 4.54–11.65 for Comorbidity vs. Absence of Diagnosis) with a very strong association in the first case and strong associations for the other classes. The Nagelkerke pseudo-*R* ^2^ values concerning the two models are reported in Table 4.

The use of ‘most likely class membership’ as a variable for further analysis may be problematic when the entropy goes much lower than 0.8 because the precision in assigning class membership is less than optimal [30]. As in our model entropy was 0.77, we repeated analysis by directly introducing the categorical outcome (i.e. diagnostic status) in the LCA 4-classes model [31], and the risk of having two or more diagnoses was identified for each latent class. Results confirmed that the Comorbidity condition is more likely for subjects in the Severe Dysregulated class (64.3%), followed by Internalizing class (51.1%), Attention/Hyperactivity class (24.9%) and, finally, by Low Problems class (7.9%). Moreover, the probability of having two or more diagnoses with respect to one diagnosis is significantly higher for individuals in Severe Dysregulated class with respect to individuals in Attention/Hyperactivity and Low Problems classes (respectively, OR = 5.42; 95% CI 2.55–11.53; OR = 21.12; 95% CI 7.62–58.59). Instead, subjects in Internalizing class have a probability only moderately lower (but significant) than those in Severe Dysregulated class to have two diagnoses (OR = 0.58; 95% CI 0.21–1.635).

## Discussion

In the present study, we examined whether different profiles of psychopathology could be identified using a person-centred statistical approach in a large and inclusive sample of Italian children and adolescents combining general-population and referred subjects. Moreover, we investigated the relationship between these profiles and DSM-IV diagnoses as well as the comorbidity of diagnoses.

Latent class analysis performed on CBCL/6–18 syndrome scales for our samples identified 4 classes. One class grouped subjects without a significant risk of elevation on any scales. A second class was characterized by children and adolescents with a high risk of being in the clinical range for Attention problems. The third class grouped subjects with a high risk of elevation on the Anxious/depressed scale. Finally, the last and more compromised class included subjects with an elevated probability of being in the clinical range for all CBCL scales, with the exception of Somatic complaints and Rule-breaking behaviour. These results are very similar to those presented by Connell et al. [11] and by Basten et al. [12]. Considering the differences between the version of the checklist used (1.5–5 vs. 6–18), the overlap between the macro-structure of our classes and the one identified by these authors appears clearly. Furthermore, observing the similarities between the profiles identified in early childhood and those identified in children and adolescents, we might guess that this way of grouping on the basis of psychopathological traits is so strong that it is independent of the developmental stage. It would be of interest to use the same approach on the Adult Behaviour Checklist 18–59 (ABCL/18–59) [32] to check whether these profiles emerge again. Finally, considering that sample features may influence the structure and the prevalence of identified classes [33], our results seem to be reliable as they were obtained from a large and heterogeneous sample, including both referred subjects and general-population subjects. Furthermore, these results are also supported by the observation that the same structure of classes has been obtained by performing separate LCAs on the two sub-samples, which yielded a substantial classification agreement.

The second significant result was the identification of a profile characterized by elevated levels of dysregulation, which was similar to previously published works. De Caluwé et al. [13] identified a higher number of classes (six), but it is worth noting that their ‘No symptoms’ class and ‘CBCL-DP’ class appear very similar to the classes identified in this work. Looking at the profile of dysregulation, we can note that in both cases it was primarily characterized by a very elevated probability of being in the clinical range for the Anxious/depressed, Attention problems, and Aggressive behaviour scales, and secondly in the other scales with the exception of Somatic complaints and, only in our sample, of Rule-breaking behaviour scale. We believe that our study confirms the existence of a dysregulated profile, with limited differences linked to the substantial differences in sample size.

The term Dysregulation Profile (DP) usually refers to the specific profile identified by Althoff [6, 34], which is characterized by the elevation of the three scales, Anxious/depressed, Attention problems, and Aggressive behaviour. In our study, we identified a larger profile of dysregulation that included the three scales used by Althoff as well as the Withdrawn/depressed, Social problems, and Thought problems scales. A possible explanation for this difference is that Althoff et al. identified the DP by performing an LCA specifically on the items of only these three scales, while our analysis was conducted on a broader spectrum of emotional and behavioural problems, including all scales of the CBCL/6–18. Our results were probably more realistic, as they were not based on an a priori hypothesis, but rather on empirical findings. Moreover, these results are not in contrast with those of Althoff. On the contrary, they confirm and extend Althoff’s results, particularly if we consider that our profile had a frequency of 7.82% in our sample. This was consistent with other studies that used LCAs and identified a prevalence of DP ranging from 4 to 8% [4, 6, 34]. Finally, there is no evidence that children with DP have elevations only in these three scales. On the contrary, several studies have claimed that these children show elevations in other scales [7, 35–37]. In conclusion, we can say that our study provides further evidence of the replicability of DP in different countries, samples, and methodologies [38].

In the second step of our study, we highlighted the diagnostic sensibility of each class. For all diagnostic categories, there was a very low percentage of subjects in the LOW class (between 4 and 20%, approximately). In the INT class, the higher percentages of diagnosis were for Anxiety Disorders (42.8%) and Mood disorders (26.4%). In the ADHD class, about half of the subjects (49.0%) had a diagnosis of Attention-Deficit/Hyperactivity Disorder, but we also found that 23.8% of subjects had an Anxiety Disorder. This result is not surprising considering that Anxiety Disorders in both children and adults are among the disorders that most commonly co-occur with Attention-Deficit/Hyperactivity Disorder [39]. Finally, in the DYS class the highest percentages we found were for Anxiety Disorders (54.3%), Mood Disorders (44.5%), and Attention-Deficit/Hyperactivity Disorder (30.7%), underlining and confirming previous results suggesting a high heterogeneity of diagnoses assigned to individuals with emotional and behavioural dysregulation [8, 34, 40].

Finally, when we analysed how the four classes were able to predict three different diagnostic profiles (absence of diagnosis vs. one diagnosis vs. two or more diagnosis), we found that the LOW class better identified an absence of diagnosis, while all three other classes were able to predict the presence of comorbidity, with the DYS class being the significantly stronger predictor as reported by other studies [41, 42]. The same results were confirmed when analyses were repeated with a more conservative approach (i.e. by incorporating outcomes into the latent class model) to correctly account for uncertainty in class membership.

There are several limitations in this study. First, it is based only on parent reports (CBCL/6–18). An interesting future direction would be to consider the CBCL not only being completed by the parents but also the Youth Self Report (YSR/11–18) [1] completed by the child/adolescent or the Teacher’s Report Form (TRF/6–18) [1] filled out by teachers. Second, the incidence of certain diagnostic outcomes was low, necessitating the collapse of several diagnoses into combined categories. Third, although the results are comparable with those of other studies, the participants were recruited from an Italian sample, which potentially limits the replicability of the results in other countries. Fourth, since we used a cross-sectional design, our findings provide only a static view of the classes. Thus, they do not provide information on the onset, progress, or changes in the class types and their syndromes over time.

## Conclusion

Using a person-centred statistical approaches, we were able to identify four different psychopathological profiles in a large sample including both referred and general-population children and adolescents. Membership in these groups appears to be related to different positive and negative outcomes in terms of psychopathological diagnoses. In particular, our results underline the presence of a profile characterized by severe emotional and behavioural dysregulation, which is mostly associated with the presence of multiple diagnoses. In conclusion, one of the main results of this work lies in the implementation of a bottom-up approach, thus using a *non*-a priori method, to provide further evidence of the replicability and clinical significance of Dysregulation Profile in different countries, with different methodologies and in a large and heterogeneous sample.
